# Differential expression of vascular endothelial growth factor mRNA vs protein isoform expression in human breast cancer and relationship to eIF-4E.

**DOI:** 10.1038/bjc.1998.356

**Published:** 1998-06

**Authors:** P. A. Scott, K. Smith, R. Poulsom, A. De Benedetti, R. Bicknell, A. L. Harris

**Affiliations:** Imperial Cancer Research Fund, Institute of Molecular Medicine, John Radcliffe, Oxford, UK.

## Abstract

**Images:**


British Journal of Cancer (1998) 77(12), 2120–2128
© 1998 Cancer Research Campaign

### Differential expression of vascular endothelial growth factor mRNA vs protein isoform expression in human breast cancer and relationship to eIF-4E

**PAE Scott[1], K Smith[1], R Poulsom[2], A De Benedetti[3], R Bicknell[1] and AL Harris[1]**

[1]Imperial Cancer Research Fund, Institute of Molecular Medicine, John Radcliffe, Oxford OX3 9DU, UK; [2]Imperial Cancer Research Fund, Lincoln's Inn Fields, London WC2A 3PX, UK; [3]Department of Biochemistry and Molecular Biology, Louisiana State University Medical Center, Shreveport, LA, USA

**Summary** Angiogenesis is the formation of new blood vessels from the existing vasculature. Vascular endothelial growth factor (VEGF) is an endothelium-specific angiogenic factor strongly implicated in pathological angiogenesis. In this study, the mRNA and protein expression of the four alternatively spliced VEGF isoforms (121, 165, 189 and 206 amino acids) were examined in normal and malignant breast tissues. Three VEGF transcripts were detected in both (121>165>189), whereas only VEGF165 protein was detected. The tumours expressed more VEGF mRNA ($P = 0.02$) and protein ($P < 0.0001$), with eight-fold more VEGF protein generated per mRNA unit ($P = 0.009$). To examine this further, the expression of eIF-4E, a translation initiation factor, was examined. Increased eIF-4E mRNA levels were detected in the tumours ($P < 0.0001$) that correlated with VEGF mRNA ($P = 0.0002$), implying co-regulation of these genes. VEGF mRNA expression was elevated in tumours expressing the epidermal growth factor receptor ($P < 0.01$), but there was no difference according to oestrogen receptor status ($P = 0.9$), node status ($P = 0.09$) or between differing histologies ($P = 0.4$). These data suggest that elevated VEGF protein expression, by both enhanced transcription and translation, is a potential means by which tumour angiogenesis is induced in breast carcinomas. VEGF expression is also significantly associated with factors correlating with a poor outcome, implying a role in progression of this disease.

**Keywords:** angiogenesis; vascular endothelial growth factor; eIF-4E translation; breast cancer; GAPDH

Angiogenesis is the formation of new blood vessels from the existing vasculature. It is a complex process involving degradation of the basement membrane, endothelial proliferation, migration, tube formation and the initiation of blood flow. Angiogenesis occurs in a wide range of biological events, including the female reproductive cycle, embryonic development and wound healing (Folkman and Shing, 1992). In the pathological setting, it is an important component of many diseases including rheumatoid arthritis and diabetic retinopathy, and is a critical, although not the only, step necessary for the growth and metastasis of tumours (Folkman, 1990). Under physiological conditions, angiogenesis is regulated strictly by a balance of stimulation and inhibition, but in the neoplastic state there appears to be a loss of control (Liotta et al, 1991).

Although several types of angiogenic factors, including lipids and peptides, have been identified (reviewed Folkman and Klagsbrun, 1987; Bicknell and Harris, 1991; Bouck, 1993; Scott and Harris, 1994), the focus has remained on the polypeptide growth factors. Of these, the angiogenic growth factor most strongly implicated in tumour angiogenesis is vascular endothelial growth factor (VEGF).

In addition to being the most selective endothelial mitogen known (Leung et al, 1989), VEGF elicits other effects on endothelial cells, including chemotaxis (Kock et al, 1994), increased permeability (Keck et al, 1989) and the release of proteinases such as urokinase plasminogen activator and collagenase (Pepper et al, 1992; Unemori et al, 1992).

*Received 13 July 1997*
*Revised 1 December 1997*
*Accepted 5 January 1998*

*Correspondence to:* AL Harris

VEGF expression has been identified at the mRNA or protein level in a range of malignancies, including gastrointestinal (Brown et al, 1993*a*; Hsu et al, 1995; Shiraishi et al, 1995; Takahaski et al, 1995; Warren et al, 1995; Maeda et al, 1996), renal (Brown et al, 1993*b*; Sato et al, 1994; Takahaski et al, 1994), bladder (Brown et al, 1993*b*), lung (Mattern et al, 1995), ovarian (Boocock et al, 1995; Olson et al, 1995), endometrial (Harrison-Woolrych et al, 1995), cervical (Guidi et al, 1995) and hepatocellular carcinomas (Mise et al, 1996) In human breast carcinoma, VEGF mRNA expression has been reported to be higher in tumour cells compared with normal ductal cells using in situ hybridization (Brown et al, 1995). The levels of VEGF protein have been quantitated in this malignancy and found to correlate with increased microvessel density (Toi et al, 1994; 1996) and early relapse in primary breast carcinoma (Toi et al, 1994). A recent study has demonstrated that VEGF protein levels in tumour samples are significantly higher in tumours compared with matched normal samples (Yoshiji et al, 1996).

To date, all studies of VEGF in breast carcinoma and normal tissues have examined total mRNA and protein. There are, however, four isoforms with significantly different biochemical and biological effects that are generated by alternative splicing (Houck et al, 1991; Tischer et al, 1991). The smallest isoform, VEGF121, is freely soluble and does not bind heparin. The inclusion of more cationic exons in the next two isoforms, VEGF165 and VEGF189, confers heparin-binding properties (Tischer et al, 1991; Ferrara et al, 1992) resulting in binding to the extracellular matrix after secretion (Houck et al, 1992; Park et al, 1993). This heparin-binding fragment has been shown recently to confer an increased mitogenic potency on the VEGF165 compared with the VEGF121 isoform (Keyt et al, 1996), although there are no reports on the relative potency of the VEGF189 isoform. The largest isoform, VEGF206, has only been identified in a fetal liver library and has similar properties to VEGF189 (Houck et al, 1991), but little is known about its biological relevance.

VEGF expression is regulated by a number of factors including hypoxia and hypoglycaemia in glioblastoma spheroids (Shweiki et al, 1995), and hormones in tissues from the female reproductive tract (Cullinanbove and Koos, 1993). Hypoxia, a feature of all solid tumours, is the most potent stimulus, and its induction results from a combination of increased transcription and mRNA stability. Recently, however, a post-transcriptional mechanism for VEGF regulation has been identified, acting through the enhancement of recruitment and binding of the mRNA to the ribosome. The mRNA of growth factors such as VEGF characteristically have long 5′ untranslated regions (UTRs) with complex secondary structures that render them inefficiently translated (Kevil et al, 1996). eIF-4E is a polypeptide that binds the 7-methylguanosine-containing cap of mRNA, unwinds the 5′ UTR of the target mRNA facilitating the identification of the translation start site by the ribosome. Elevated eIF-4E levels have been associated with increased growth rates and transformation of cell lines (De Benedetti and Rhoads, 1990), and increased levels of VEGF protein (Kevil et al, 1996). Increased expression of eIF-4E has been found in both breast carcinoma tissues compared with fibroadenomas (Kerekatte et al, 1995) and breast carcinoma cell lines compared with normal breast cell lines (Anthony et al, 1996) and may represent a means of regulating expression of genes including angiogenic factors and subsequent tumour growth (Kevil et al, 1996).

The aims of this study were to quantify and locate the expression of the VEGF isoforms at the mRNA and protein levels in both normal and breast carcinoma tissues. The relationship between mRNA and protein expression was studied in tumours vs normal tissues as well as the potential role of eIF-4E in breast carcinoma in regulating that expression. The levels of VEGF mRNA, protein and eIF-4E were also compared with factors known to affect prognosis such as oestrogen receptor (ER), epidermal growth factor receptor (EGFR), node status and histology of the tumours.

### MATERIALS AND METHODS

### Isolation of RNA from cells and tissues and ribonuclease protection assay (RPA)

Total RNA was prepared by acid guanidinium thiocyanate–phenol–chloroform extraction (Chomczynski and Sacchi, 1987). For the ribonuclease protection assays, radiolabelled riboprobes were synthesized with [$\alpha$-$^{32}$P]CTP (Amersham, UK) from linearized plasmid DNA using the in vitro transcription method (Ausubel et al 1987).

Two VEGF riboprobes were used: the first was designed to protect the full length of the smallest isoform (VEGF121 yielding a 471-base band, with a lower band of 427 representing the remaining isoforms). This 520-base probe was generated by linearizing the full-length cDNA for VEGF121 (including 26 bp of 3′ untranslated sequence) cloned into pBluescript SK with *Eco*RV and transcribing with T7 RNA polymerase. To determine the isoforms contributing to this second smaller band, a new construct was designed to protect its largest fragment, $VEGF_{189}$, with the remaining isoforms forming two bands lower on the gel. The cDNA coding for the mature VEGF189 protein was cloned into BS KS + in the *Xba*I and *Eco*RI sites and the probe generated using *Not*I and T3 polymerase. The largest protected fragment was 567 bases, with a 345-bp fragment formed from both the VEGF121 and the VEGF165 isoforms, and a third band of 150 bases representing VEGF165 alone. The generation of the eIF-4E riboprobe has been described elsewhere (De Benedetti et al, 1991).

RPAs were performed essentially as described (Ausubel et al, 1987) with a minimum of $10^5$ c.p.m. of each antisense riboprobe hybridized overnight at 55°C to each sample with 10 µg of transfer RNA as a negative control. The RNAase digestion of the unhybridized RNA fragments was performed the next day by adding 350 µl of RNAase digestion buffer with 40 µg ml$^{-1}$ RNAase A and 1000 units ml$^{-1}$ RNAase T1 to each sample and incubating for 30 min at room temperature. The RNAases were inactivated by adding 12.5 µl of a mixture containing 16% sodium dodecylsulphate (SDS) solution with 4 µg µl$^{-1}$ proteinase K and incubating at 37°C for 15 min. After phenol extraction and precipitation in 2 volumes of ethanol, the samples were resuspended and loaded onto a 6% polyacrylamide/urea sequencing gel followed by autoradiography.

The resulting bands were quantitated densitometrically (Bioimage densitometer, Millipore) and signals normalized to the external control. This control was formed from the hybridization of a sense and antisense riboprobe to glyceraldehyde-3-phosphate (GAPDH), with the sense riboprobe generated by linearizing the construct 30 bases beyond the end of the GAPDH sequence. This gives two protected fragments: the endogenous gene (120 bp) and the external control fragment (150 bp). Before aliquotting, each sample was run on a 1% agarose gel under RNAase-free conditions, and the RNA concentration measured spectrophotometrically, as described in Scott et al (1997).

### In situ hybridization

In situ hybridizations were performed using single-stranded RNA $^{35}$S-labelled riboprobes hybridized with 4-µm sections of formalin-fixed, paraffin-embedded tissue prepared earlier by dewaxing in xylene/0.1% DEPC then rehydrating in serial washes of 100%, 80%, 60% and 30% ethanol. The sections were then permeabilized with proteinase K (20 µg ml$^{-1}$) for 10 min, washed thoroughly in phosphate buffered saline (PBS) and the tissues acetylated with 0.1 M triethanolamine and acetic anhydride (1.25 ml in 500 ml) before dehydration in ethanol in the reverse order mentioned above, ready for hybridization. The sections were then incubated with $10^6$ c.p.m. of riboprobe in hybridization buffer (Senior et al, 1987) that had been heated to 80°C, and incubated overnight at 55°C. After washing extensively in 50% formamide at 50°C, the sections were incubated with 100 µg ml$^{-1}$ RNAase A for 1 h to remove the unhybridized riboprobe. The slides were then washed, fixed in 30% ethanol/0.3 M ammonium acetate, air dried and placed in emulsion (Senior et al, 1987). After 7 days, the slides were developed, washed and the signal visualized, and compared with Giemsa-counterstained light-field sections. A β-actin hybridization was used as a positive control.

### Extraction and analysis of tumour membranes and cytosols

Tumour membranes, cytosols and nuclei were prepared as described previously (Sacks et al, 1993). Briefly, the tumours were

© Cancer Research Campaign 1998

frozen in liquid nitrogen, ground up in a similar way as for RNA extraction before addition of an ice-cold Hepes/EDTA buffer containing proteinase inhibitors, then homogenized using a Dounce homogenizer. The homogenate was centrifuged in a bench-top centrifuge at 4°C, yielding the first fraction containing mostly DNA and other nuclear debris, and was retained as the 'nuclei' fraction. The supernatant was removed carefully and centrifuged at 100 000 *g* to bring down any membrane fractions (the 'membrane' fraction while the supernatant forms the 'cytosol' fraction).

### ELISA analysis of tissues

The VEGF protein levels were measured using ELISA (R&D Systems, Abingdon, UK). As this ELISA had not been used previously for assaying such samples, the accuracy was validated by comparing the VEGF protein standards diluted 50:50 with the sample buffer (Tris-buffered saline pH 7.5) with that provided by the manufacturer, as well as the effects of adding a constant amount of sample to the standards made up with the manufacturer's buffer. A final validation was carried out by serially diluting a sample to determine whether the detection of VEGF decreased accordingly. The study was commenced after confirming the accuracy of the ELISA under these conditions.

**Table 1** The clinicopathological characteristics of the patients whose breast carcinomas were examined for VEGF expression

| Patient characteristics | Number |
|---|---|
| Age mean (median, range) years | 55 (35–86) |
| <50 years | 18 |
| ≥50 years | 34 |
| Lymph nodes | |
| Positive/negative | 15/37 |
| Histology | |
| Ductal | 41 |
| Lobular | 7 |
| Other | 4 |
| ER[a] (median, range) | (1.6–742) |
| <10 | 15 |
| ≥10 | 37 |
| EGFR[a] (median, range) | 13 (0–77) |
| <20 | 32 |
| ≥20 | 20 |

[a]fmol $mg^{-1}$ protein.

**Table 2** The numbers of cases examined and levels of VEGF mRNA and protein expression in normal and breast carcinoma tissues. The VEGF mRNA was standardized against an external control, whereas the VEGF protein was measured as pg per 100 μg total protein for each sample

| | VEGF mRNA (standardized to external control) | | VEGF protein (pg per 100 μg total protein) | |
|---|---|---|---|---|
| Sample | Normal | Tumour | Normal | Tumour |
| Number | 16 | 46 | 15 | 38 |
| Mean ± s.e.m. | 8.9 ± 2.3 | 18.6 ± 2.6 | 21.9 ± 4.1 | 277 ± 68 |
| Median | 5.9 | 12.8 | 19 | 113 |
| Min–max | 0.7–37 | 1.2–8 | 2.8–49 | 6–2305 |

### Immunoblotting analysis of tissues

The protein samples were separated by sodium dodecyl sulphate polyacrylamide gel electrophoresis (SDS–PAGE) and transferred onto a PVDF membrane at 4°C at 15 V overnight. The membrane was then blocked for a minimum of 2 h in blocking buffer (PBS, 0.1% Tween 20, 5% Marvel fat-free milk) before incubation with the anti-VEGF rabbit polyclonal antibodies (Zhang et al, 1995) diluted 1:200 in blocking buffer. After washing, the membrane was incubated for 30 min with [$^{125}$I]protein A (Amersham, UK), washed and then exposed directly to radiographic film at –80°C.

### Statistics

All descriptive and non-parametric statistics were performed using Statview 4.5 (Abacus Concepts, Berkeley, CA, USA). The comparative statistical analysis was calculated using the Mann–Whitney *U*-test, whereas the correlations were calculated using the Spearman rank correlation. The mean values are presented with the standard error of the mean.

### RESULTS

### VEGF mRNA expression in human breast carcinoma and normal tissues

Fifty-two cases of breast carcinoma were examined for expression of either VEGF mRNA and/or protein (see Table 1 for clinicopathological details). VEGF mRNA expression was examined in 46 cases, together with matched samples of normal breast tissues resected during mastectomy from 11 of those 46 cases, one case of unmatched normal tissues and four samples of normal tissues from breast reduction specimens (Table 2). All tumour and normal samples expressed the isoforms in order of decreasing abundance, 121>165>189, except for one tumour in which the pattern of expression of VEGF was 165>121>189. Although the VEGF 121 isoform was the most abundant species in most cases, there was considerable variation in the extent to which it predominated, ranging from 41% to 83% of the total VEGF message. In addition, a band of approximately 280 bases was detected in 36 cases of breast carcinoma but not in any of the normal breast tissues.

When measuring the levels of VEGF mRNA, initially GAPDH levels were considered as an internal control. However, levels of this enzyme fluctuated sixfold when compared with an external spiked control; when both VEGF mRNA and GAPDH mRNA expression was standardized against an external spiked control, there was a significant correlation between their expression levels ($r = 0.77$, $P < 0.0001$, Spearman rank coefficient test). Thus, thereafter, an external spiked control was used to standardize the densitometric signals between samples.

The levels of VEGF mRNA, standardized to an external control, varied up to 70-fold across the tumours ($n = 46$, mean 18.6 ± 2.6), 20-fold across the normal tissues resected from mastectomy specimens ($n = 12$, mean 9 ± 3) and two-fold across the normal tissues from mammoplasty tissues ($n = 4$, mean 8.5 ± 1.7) (Table 2). There was a significant difference between the levels of VEGF mRNA in the tumour compared with all the normal samples ($P = 0.02$). There was also a significant difference when the reduction mammoplasty samples were excluded, that is only the normal tissues taken from regions adjacent to the tumours were included in the analysis ($P = 0.03$). When the levels of VEGF mRNA in

© Cancer Research Campaign 1998

**Figure 1** An RNAase protection demonstrating increased expression of both eIF-4E and VEGF mRNA in the tumour samples from three cases with matching normal samples, as well as low levels of expression in four normal samples taken from reduction mammoplasties. The control used was formed from the hybridization of sense and antisense GAPDH riboprobes added to each sample normal breast and carcinoma tissues taken from the same patient were compared, the VEGF mRNA expression was higher in 9 out of 11 cases ($n$ = 11; normal mean = 9 ± 3.3; tumour mean = 15.6 ± 5, $P$ = 0.09), but this was not significant, probably reflecting the smaller numbers available for direct comparison (see Figure 1).

The VEGF mRNA expression in EGFR-positive tumours ($n$ = 17; mean 28.6 ± 5.8) was significantly higher than the EGFR-negative cases ($n$ = 29; mean = 13.2 ± 2.3; $P < 0.01$). There was no significant difference between VEGF mRNA expression in cases negative or positive for ER status ($P$ = 0.09), node status ($P$ = 0.9) or between differing histologies ($P$ = 0.4; $n$ = 39 ductal, five lobular and two other histology subtypes).

### The distribution of VEGF mRNA in breast carcinoma

To determine which cells were expressing the VEGF mRNA, formalin-fixed, paraffin-embedded samples of tumour and normal breast were analysed by in situ hybridization. The linearized VEGF121 template was used to generate riboprobes for this experiment.

The highest levels of VEGF mRNA were detected in the epithelial cells in both normal and malignant tissues (Figure 2), with the latter expressing higher levels than their normal counterparts. Low levels of VEGF mRNA were detected in the stromal cells of both tumour and normal tissues.

### VEGF protein expression in human breast carcinoma and normal tissues

The protein levels in the tumours and normal tissue, the latter all from mastectomy specimens, were examined using ELISA (R & D Systems, UK). Having validated the ELISA for use with these samples (see Materials and methods), the subcellular localization of VEGF in samples from the membrane, cytosolic and nuclear fractions of eight tumours and six normal samples taken from mastectomy specimens were examined. In all of the tumour samples examined using ELISA, the highest VEGF concentration (pg per 100 μg total protein) was found in the membrane fraction (see Table 3), followed by the cytosolic fraction with lower levels in the nuclear fraction. The membrane VEGF concentration (mean 873 ± 261) was significantly higher than the nuclear fraction (mean 367 ± 165) in the tumour samples ($n$ = 8, $P$ = 0.04) but not the cytosolic fraction (mean 503 ± 156; $P$ = 0.2). In the normal samples, the VEGF concentration highest in the membrane fraction (mean 35.5 ± 5.4), which was significantly higher than the cytosol fraction (cytosol mean 17.2 ± 2.7; $n$ = 6; $P$ = 0.02) but not the nuclear fraction ($n$ = 6, mean 22.7 ± 3.6; $P$ = 0.07). Thus, the membrane fraction was used to measure the levels of VEGF protein in a further 33 tumour and 13 normal samples. In 13 normal samples, the corresponding tumour tissues from the same patient were available for comparison.

**Figure 2** In situ hybridization with light (**A** and **C**) and dark (**B** and **D**) fields demonstrating VEGF expression predominantly in the epithelial component of both normal (**A** and **B**) and breast carcinoma tissues (**C** and **D**)

**Table 3** The subcellular localization of VEGF protein in normal and malignant breast tissues

| | VEGF protein (pg per 100 μg total) | |
|---|---|---|
| **Tissue fraction** | **Normal ($n$ = 6)** | **Tumour ($n$ = 8)** |
| Membrane | 35.5 ± 5.4 | 873 ± 261 |
| Cytosolic | 17.2 ± 2.7 | 503 ± 156 |
| Nuclear | 22.7 ± 3.6 | 367 ± 165 |

**Table 4** The amount of protein generated per unit of mRNA in normal and breast carcinoma tissues

| | pg protein per unit mRNA | |
|---|---|---|
| **Sample** | **Normal** | **Tumour** |
| Number | 13 | 13 |
| Mean±s.e.m. | 17.9 ± 3.2 | 144 ± 17.9 |
| Median | 16.8 | 80 |
| Min–max | 5.6–32.5 | 14.6–395 |

**Figure 4** The correlation of VEGF mRNA and protein in the breast carcinoma cases ($r$ = 0.6, $P$ < 0.0007). After measurement by densitometry, the mRNA was standardized against an external control, whereas the protein is expressed as pg VEGF per 100 μg total protein for each sample

**Figure 3** The amount of VEGF (pg per 100 μg total protein) generated per unit RNA in normal vs tumour samples taken from the same patients. □, pg VEGF unit RNA normal; ■, pg VEGF unit RNA tumour

**Figure 5** The detection of VEGF165 protein in five breast carcinoma samples (lanes 3–7) by immunoblotting using a rabbit polyclonal antibody to VEGF. The characteristic doublet seen under reducing conditions (due to unequal glycosylation) is the same size as the VEGF165 control (lane 2) and larger than the VEGF121 protein from conditioned media of transfected cells (lane 1), with the molecular weight marker sizes indicated at the left. An aliquot (100 μg) of total protein was loaded for each tumour sample for comparison with 1 μg of recombinant VEGF165

The VEGF protein expression in the normal samples ($n$ = 15, mean 21.9 ± 4.1) differed significantly from that in the tumour samples ($n$ = 38, mean 277 ± 68, $P$ < 0.0001) (see Table 2). Furthermore, there was up to sixfold (mean 3.6 ± 0.6) more protein generated per unit mRNA in the 13 matched tumours (mean protein concentration 161 ± 40) compared with the corresponding normal samples (mean protein concentration 15.8 ± 2.8, $P$ < 0.0001) (see Table 4 and Figure 3).

There was a strong correlation between the tumour VEGF mRNA and protein levels ($n$ = 38, mRNA mean 18.6 ± 2.6, protein mean 277 ± 68, $r$ = 0.6, $P$ < 0.0007) (Figure 4). There was no significant difference in VEGF protein expression in cases positive or negative for EGFR ($P$ = 0.62), ER expression ($P$ = 0.3), node involvement ($P$ = 0.56) or between differing histologies ($P$ = 0.37).

### VEGF isoform expression in normal and breast carcinoma samples

VEGF165 was the only isoform detected in nine carcinoma and two normal samples using immunoblotting. As with the ELISA, the expression was detected predominantly in the membrane fraction, with some also detected in the cytosolic fraction of samples expressing high levels of VEGF overall. The VEGF protein is detected as a doublet under reducing conditions because of differential glycosylation (Ferrara et al, 1992) (Figure 5) and was the same size as the recombinant VEGF165 doublet. Although detectable in the membrane fractions of xenografts formed from MCF-7 cells transfected with VEGF121, neither this isoform nor VEGF189 was detected in the human breast carcinoma samples.

### The expression of eIF-4E in normal and breast carcinoma tissues eIF-4E mRNA levels were measured in three normal samples from mastectomy cases, four reduction mammoplasty samples and 44 breast carcinoma cases and standardized against the spiked external control. eIF-4E mRNA expression was detected in two reduction mammoplasty samples, with low levels in the normal tissues from mastectomies. In the three paired normal and tumour samples, the levels of eIF-4E mRNA were between four and

© Cancer Research Campaign 1998

**Figure 6** The correlation between eIF-4E and VEGF mRNA expression in the 38 tumours examined for both factors ($r = 0.58$, $P = 0.0002$). Each was measured by densitometry and standardized against a spiked external control

42-fold higher in the tumours. When the levels of all of the normal tissues ($n = 7$, normal mean $0.5 \pm 0.22$, median 0.1) were compared with the tumour tissues examined ($n = 42$, tumour mean $6.8 \pm 0.8$, median 4.8), there was a significant difference ($P < 0.0001$).

In the cases in which both tumour eIF-4E mRNA (mean $6.8 \pm 0.8$) and VEGF mRNA expression (mean $19.3 \pm 2.7$) were examined there was a strong correlation ($n = 42$, $r = 0.58$, $P = 0.0002$) (see Figure 6), but not between eIF-4E mRNA and VEGF protein expression ($r = 0.07$, $P = 0.98$).

### DISCUSSION

VEGF mRNA and protein levels correlate with increased microvascular density and a poor prognosis in breast carcinoma (Toi et al, 1994). High vascular counts in breast tumours as an index of angiogenesis are associated with a significant reduction in survival (Horak et al, 1992; Weidner et al, 1992; Fox et al, 1994), and thus it is likely that VEGF is partially responsible for breast cancer angiogenesis.

The findings in this study of VEGF mRNA and protein in all samples analysed are in contrast with one report (Toi et al, 1995) and in agreement with another (Yoshiji et al, 1996). The latter study reported higher VEGF mRNA in 18 breast carcinomas compared with normal breast tissues from the same patients, and detected VEGF immunohistochemically only in the tumour sections. In this study, the mRNA levels in 46 cases and 16 normal samples (11 matched normal tissues, one unmatched and four breast reduction samples) were quantitated using ribonuclease protection assays. The protein levels in 38 cases were measured using ELISA and compared with 13 matched normal samples and two unmatched normal tissues from mastectomies. In addition to there being significant differences between the VEGF expression by tumours and the normal samples at both the mRNA and the protein levels, there was a strong correlation between the mRNA and protein expression. Furthermore, although there was a correlation between mRNA and protein expression, the amount of protein detected in normal tissues was several fold lower than in the tumours for a given level of mRNA. This suggests that VEGF expression is modified post-transcriptionally in tumours compared with normal breast tissue.

There are several possible explanations for this difference. Elevated levels of eIF-4E protein had been reported in breast carcinoma cell lines (Anthony and De Benedetti, 1996), and in one previous report in which the expression in 38 breast carcinomas was compared with fibroadenomas or breast reduction specimens (Kerekatte et al, 1995). The same number of tumours was examined in this study, but, in addition, the eIF-4E expression in tumours was compared with normal tissues from the same patient, as well as samples from breast reductions. The eIF-4E mRNA levels were up to 40-fold higher in the tumour samples compared with the paired normal samples. Consistent with the findings of others examining similar samples for eIF-4E protein expression (Kerekatte et al, 1995), the eIF-4E mRNA levels in reduction mammoplasty samples were very low in two samples and undetectable in two samples. In agreement with the previous report, there was no correlation with ER status (Kerekatte et al, 1995), nor with EGFR, node status, nor histology.

There was a strong correlation between tumour VEGF mRNA and eIF-4E mRNA levels. As eIF-4E does not regulate VEGF mRNA transcription, this suggests that a common factor regulates both eIF-4E and VEGF mRNA expression resulting in a marked elevation of VEGF protein levels. eIF-4E expression is regulated in response to growth induction by c-myc (Rosenwald et al, 1993) and eIF-4E phosphorylation is regulated by overexpression of ornithine decarboxylase (Shimogori et al, 1996) that is, in turn, regulated when eIF-4E is overexpressed (Shantz et al, 1996). Recent data have shown that mutant p53 protein regulates VEGF mRNA expression (Kieser et al, 1994), suggesting a link with tumour progression, and it would be of interest to investigate whether mutant p53 protein also regulates eIF-4E.

There was no correlation between VEGF protein and eIF-4E mRNA levels in the tumours examined. Although eIF-4E protein is reported to correlate with the mRNA levels (Rosenwald et al, 1993; Anthony and De Benedetti, 1996), the eIF-4E protein has a short half-life compared with the mRNA, is subject to phosphorylation, and therefore the percentage of functional translation initiation factor protein may differ between tumour samples. It is also possible that eIF-4E represents just one mechanism regulating post-transcriptional VEGF expression in malignancy. Other mechanisms include stabilization of the VEGF protein, as when bound to $\alpha_2$-macroglobulin (Soker et al, 1993).

The VEGF mRNA levels were significantly higher in EGFR-positive tumours. EGF stimulation of EGFR has been reported to induce VEGF expression in glioblastomas, thereby inducing angiogenesis and resulting in their characteristic vascular appearance (Goldman et al, 1993), and a similar mechanism may operate in these breast carcinomas. EGFR expression (Nicholson et al, 1988) and increased microvascular density have both been associated with a poor prognosis in breast cancer patients (Fox et al, 1994). The elevated VEGF is likely to contribute to the increased vessel density, and the association with a poorer prognosis implicates VEGF further as a major angiogenic factor contributing to tumour growth and metastasis.

When measuring the VEGF protein, the highest levels were found in the membrane fraction, with lower levels in the cytosols. VEGF was detected in the nuclear fraction, which may reflect minor contamination of this fraction during preparation and/or translocation of VEGF into the nucleus (Moroianu 1994). It is less likely to represent artefact as there was a wide variation in expression in the nuclear fractions prepared from different tumours, with no relationship to total cellular VEGF protein levels. Only VEGF165 was detectable using immunoblotting, despite detection of VEGF121, VEGF165

© Cancer Research Campaign 1998

and VEGF189 transcripts. The VEGF121 protein may be difficult to detect because it is freely soluble and may bind and be internalized soon after secretion; its detection in the VEGF121 transfectant xenografts, however, confirmed that it can be detected if sufficiently abundant. It is possible, therefore, that antagonists such as suramin could be used to block the angiogenesis induced by the heparin-binding VEGF165 isoform (Braddock et al, 1994).

The extent to which the VEGF121 mRNA isoform predominated varied from 41% to 83%, with VEGF165 making up most of the remaining VEGF signal. VEGF189 was detected in all the samples but at a much lower level. In addition to the recognized isoforms, an additional band was detected that may correspond to another splice variant. This was most likely to be the 267-bp fragment described in glioblastomas (Berkman et al, 1993) as it was also detected in the glioblastoma cell line used as a loading control.

In determining the levels of mRNA, several internal and external controls to ensure even loading were assessed. These included the glycolytic enzyme, GAPDH, as an internal control, and the combination of GAPDH sense and antisense probes as an external control. The latter proved to be better as it is not possible to rely upon a gene used as an internal control being expressed equally in all of the samples. In this study, when standardized against an external control, there was a sixfold fluctuation in the levels of the commonly used housekeeping gene, GAPDH, and also a strong significant correlation between GAPDH and VEGF mRNA levels. This may be explained by a factor such as hypoxia, which is present in all solid tumours and which is known to up-regulate expression of both genes (Shweiki et al, 1992; Graven et al, 1994). GAPDH is also up-regulated during proliferation (Meyer et al, 1992; Mansur et al, 1993), which may explain the elevated levels in some tumours and the lower expression in normal tissues. The co-existence of increased VEGF and GAPDH mRNA expression may indicate a more metabolically active tissue. Given the correlation between VEGF mRNA levels and a poorer prognosis in breast carcinoma (Relf et al, 1997), an independent comparison of GAPDH and prognosis is warranted.

In summary, significantly elevated levels of VEGF mRNA and protein have been demonstrated in this study in breast carcinomas compared with normal tissues. The cells responsible for this enhanced VEGF expression are the tumour cells, with lower levels of expression by the stromal elements. The VEGF mRNA levels were higher in EGFR-positive cases and correlated with eIF-4E and GAPDH mRNA expression. There was a strong correlation between VEGF mRNA and protein expression. This suggests that VEGF expression in breast carcinoma is controlled by a combination of transcriptional regulation by factors such as hypoxia and EGF, and translational regulation by factors such as eIF-4E. Each factor may itself be subject to regulation: eIF-4E mRNA levels are regulated by hypoxia (unpublished observation), whereas the activity of the protein is modulated by phosphorylation (Rhoads, 1993).

The greatest difference between tumour and normal breast tissues were amounts of VEGF protein expressed by each. This emphasizes the potential importance of factors such as eIF-4E in tumorigenesis in a common cancer. The role of this transcription initiation factor in malignancy is beginning to emerge, and mechanisms that regulate its expression may be of therapeutic interest.

### ACKNOWLEDGEMENTS

Prudence Scott has been in receipt of grants from, and gratefully acknowledges the support of, the Rhodes Trust, the Imperial Cancer Research Fund, the Health Research Council of New Zealand, The Federation of University Women and the Nuffield Dominions Trust.

### REFERENCES

Anthony B, Carter P and De Benedetti A (1996) Overexpression of the proto-oncogene/translation factor 4E in breast carcinoma cell lines. *Int J Cancer* **65**: 858–863

Ausubel FM, Brent R, Kingston RE, Moore DD, Seidman JG, Smith JA and Struhl K (1987) *Current Protocols in Molecular Biology*. John Wiley & Sons: New York

Berkman RA, Merrill MJ, Reinhold WC, Monacci WT, Saxena A, Clark WC, Robertson JT, Ali IU and Oldfield EH (1993) Expression of the vascular permeability factor/vascular endothelial growth factor gene in central nervous system neoplasms. *J Clin Invest* **91**: 153–159

Bicknell R and Harris AL (1991) Novel growth regulatory factors and tumour angiogenesis. *Eur J Cancer* **27**: 781–785

Boocock CA, Charnockjones DS, Sharkey AM, Mclaren AM, Barker PJ, Wright KA, Twentyman PR and Smith SK (1995) Expression of vascular endothelial growth-factor and its receptors flt and kdr in ovarian-carcinoma. *J Natl Cancer Inst* **87**: 506–516

Bouck N (1993) Angiogenesis: a mechanism by which oncogenes and tumor suppressor genes regulate. In *Oncogenes and Tumor Suppressor Genes in Human Malignancies*, Benz CC and Liu ET (eds) pp. 359–371. Kluwer Academic Publishers: Boston

Braddock PS, Hu DE, Fan TPD, Stratford IJ, Harris AL and Bicknell R (1994) A structure-activity analysis of antagonism of the growth factor and angiogenic activity of basic fibroblast growth factor by suramin and related polyanions. *Br J Cancer* **69**: 890–898

Brown LF, Berse B, Jackman RW, Tognazzi K, Manseau EJ, Senger DR and Dvorak HF (1993*a*) Expression of vascular permeability factor (vascular endothelial growth factor) and its receptors in adenocarcinomas of the gastrointestinal tract. *Cancer Res* **53**: 4727–4735

Brown LF, Berse B, Jackman RW, Tognazzi K, Manseau EJ, Dvorak HF and Senger DR (1993*b*) Increased expression of vascular permeability factor (vascular endothelial growth factor) and its receptors in kidney and bladder carcinomas. *Am J Pathol* **143**: 1255–1262

Brown LF, Berse B, Jackman RW, Tognazzi K, Guidi AJ, Dvorak HF, Senger DR, Connolly JL and Schnitt SJ (1995) Expression of vascular permeability factor (vascular endothelial growth factor) and its receptors in breast cancer. *Hum Pathol* **26**: 86–91

Chomczynski P and Sachi N (1987) Single-step method of RNA isolation by acid guanidinium thiocyanate–phenol–chloroform extraction. *Anal Biochem* **162**: 156–159

Cullinanbove K and Koos RD (1993) Vascular endothelial growth-factor vascular-permeability factor expression in the rat uterus – rapid stimulation by estrogen correlates with estrogen-induced increases in uterine capillary-permeability and growth. *Endocrinology* **133**: 829–837

De Benedetti A and Rhoads RE (1990) Overexpression of eukaryotic protein synthesis initiation factor 4E in HeLa cells results in aberrant growth and morphology. *Proc Natl Acad Sci USA* **87**: 8212–8216

De Benedetti AS, Joshi-Barve S, Rinker-Schaeffer C and Rhoads RE (1991) Expression of antisense RNA against initiation factor eIF-4E mRNA in HeLa cells results in lengthened cell division times, diminished translation rates, and reduced levels of both eIF-4E and the p220 component of eIF-4. *Mol Cell Biol* **11**: 5435–5445

Ferrara N, Houck K, Jakeman L and Leung DW (1992) Molecular and biological properties of the vascular endothelial growth factor family of proteins. *Endocrinol Rev* **13**: 18–32

Folkman J (1990) What is the evidence that tumors are angiogenesis dependent? *J Natl Cancer Inst* **82**: 4–6

Folkman J and Klagsbrun M (1987) Angiogenic factors. *Science* **235**: 442–447

Folkman J and Shing J (1992) Angiogenesis. *J Biol Chem* **2**: 10931–10934

Fox S, Leek R, Smith K, Hollyer J, Grenall M and Harris A (1994) Tumor angiogenesis in node negative breast carcinomas–relationship to epidermal growth factor receptor and survival. *Breast Cancer Res Treat* **29**: 109–116

Goldman CK, Kim J, Wong WL, King V, Brock T and Gillespie GY (1993) Epidermal growth factor stimulates vascular endothelial growth factor production by human malignant glioma cells: a model of glioblastoma multiforme pathophysiology. *Mol Biol Cell* **4**: 121–33

Graven KK, Troxler RF, Kornfeld H, Panchenko MV and Farber HW (1994) Regulation of endothelial cell glyceraldehyde-3-phosphate dehydrogenase expression by hypoxia. *J Biol Chem* **269**: 24446–24453

© Cancer Research Campaign 1998

Guidi AJ, Abu-Jawdeh G, Berse B, Jackman RW, Tognazzi K, Dvorak HF and Brown LF (1995) Vascular permeability factor (vascular endothelial growth factor) expression and angiogenesis in cervical neoplasia. *J Natl Cancer Inst* **87**: 1237–1245

Harrison-Woolrych ML, Sarkey AM, Charnockjones DS and Smith SK (1995) Localization and quantification of vascular endothelial growth-factor messenger-ribonucleic-acid in human myometrium and leiomyomata. *J Clin Endocrinol Metab* **80**: 1853–1858

Horak ER, Leek R, Lkenk N, LeJeune S, Smith K, Stuart N, Greenall M, Strepniewska K and Harris AL (1992) Angiogenesis, assessed by platelet/endothelial cell adhesion molecule antibodies, as indicator of node metastases and survival in breast cancer. *Lancet* **340**: 1120–1124

Houck KA, Ferrara N, Winer J, Cachianes G, Li B and Leung DW (1991) The vascular endothelial growth factor family: identification of a fourth molecular species and characterization of alternative splicing of RNA. *Mol Endocrinol* **5**: 1806–1814

Houck KA, Leung DW, Rowland AM, Winer J and Ferrara N (1992) Dual regulation of vascular endothelial growth factor bioavailability by genetic and proteolytic mechanisms. *J Biol Chem* **267**: 26031–26037

Hsu S, Huang F and Friedman E (1995) Platelet-derived growth factor-b increases colon-cancer cell-growth in-vivo by a paracrine effect. *J Cell Physiol* **165**: 239–245

Keck PJ, Hauser SD, Krivi G, Sanzo D, Warren, Feder J and Connolly DT (1989) Vascular permeability factor, an endothelial cell mitogen related to PDGF. *Science* **246**: 1309–1312

Kerekatte V, Smiley K, Hu B, Smith A, Gelder F and De Benedetti A (1995) The proto-oncogene/translation factor eIF-4E: a survey of its expression in breast carcinomas. *Int J Cancer* **64**: 27–31

Kevil CH, De Benedetti A, Payne DK, Coe LL, Laroux FS and Alexander JS (1996) Translational regulation of vascular permeability factor by eukaryotic initiation factor 4E: implications for tumor angiogenesis. *Int J Cancer* **65**: 785–790

Keyt BA, Berleua LT, Nguyen HV, Chen H, Heinsohn H, Vandlen R and Ferrara N (1996) The carboxyl-terminal domain (111–165) of vascular endothelial growth factor is critical for its mitogenic potency. *J Biol Chem* **271**: 7788–7795

Kieser A, Weich HA, Brandner G, Marme D and Kolch W (1994) Mutant p53 potentiates protein kinase C induction of vascular endothelial growth factor expression. *Oncogene* **9**: 963–969

Koch AE, Harlow LA, Haines GK, Amento EP, Unemori EN, Wong WL, Pope RM and Ferrara N (1994) Vascular endothelial growth factor. A cytokine modulating endothelial function in rheumatoid arthritis. *J Immunol* **152**: 4149–4156

Leung DW, Cachianes G, Kuang WJ, Goeddel DV and Ferrara N (1989) Vascular endothelial growth factor is a secreted angiogenic mitogen. *Science* **246**: 1306–1309

Liotta LA, Steeg PS and Stetler-Stevenson WG (1991) Cancer metastasis and angiogenesis: an imbalance of positive and negative regulation. *Cell* **64**: 327–336

Maeda K, Chung YS, Ogawa Y, Takatsuka S, Kang SM, Ogawa M, Sawada T and Sowa M (1996) Prognostic value of vascular endothelial growth-factor expression in gastric-carcinoma. *Cancer* **77**: 858–863

Mansur NR, Meer SK, Wurzer JC and Sirover MA (1993) Cell cycle regulation of the glyceraldehyde-3-phosphate dehydrogenase/uracil DNA glycosylase gene in normal human cell. *Nucleic Acids Res* **21**: 993–998

Mattern J, Koomagi R and Volm M (1995) Vascular endothelial growth-factor expression and angiogenesis in nonsmall cell lung carcinomas. *Int J Oncol* **6**: 1059–1062

Meyer SK, Rahman MN, Wurzer JC and Sirover MA (1992) Proliferative dependent regulation of the glyceraldehyde-3-phosphate dehydrogenase-uracil DNA glycosylase gene in human cells. *Carcinogenesis* **13**: 2127–2132

Mise M, Arii S, Higashituju H, Furutani M, Nuwano M, Harada T, Ishigami SI, Toda Y, Nakayama H, Fujumoto M, Fujita J and Imamura M (1996) Clinical-significance of vascular endothelial growth-factor and basic fibroblast growth-factor gene-expression in liver turner. *Hepatology* **23**: 455–464

Moroianu J (1994) Nuclear translocation of angiogenin in proliferating endothelial cells is essential to its angiogenic activity. *Proc Natl Acad Sci USA* **91**: 1677–1681

Nicholson S, Sainsbury JRC, Needham GK, Chambers P, Farndon JR and Harris AL (1988) Quantitative assays of epidermal growth factor in human breast cancer: cut off points of clinical relevance. *Int J Cancer* **42**: 36–41

Olson TA, Mohanraj D and Ramakrishnan S (1995) The selective-inhibition of vascular-permeability factor (vpf) expression in ovarian-carcinoma cell-lines by gonadotropin-releasing-hormone (gnrh) agonist. *Int J Oncol* **6**: 905–910

Park JE, Keller GA and Ferrara N (1993) Vascular endothelial growth-factor (vegf) isoforms – differential deposition into the subepithelial extracellular-matrix and bioactivity of extracellular matrix-bound vegf. *Mol Biol Cell* **4**: 1317–1326

Pepper MS, Ferrara N, Orci L and Monesano R (1992) Potent synergism between vascular endothelial growth factor and basic fibroblast growth factor in the induction of angiogenesis in vitro. *Biochem Biophys Res Commun* **189**: 824–831

Rosenwald IB, Rhoads DB, Callana LD, Isselbacher KJ and Schmidt EV (1993) Increased expression of the eukaryotic translation initiation factors eIF-4E and eIF-2a in response to growth induction by *c-myc*. *Proc Natl Acad Sci USA* **90**: 6175–6178

Relf M, Le Jeune S, Scott PAE, Fox S, Smith K, Leek R, Moghaddam A, Whitehouse R, Bicknell R and Harris AL (1997) Expression of the angiogenic factors vascular endothelial-cell growth factor, acidic and basic fibroblast growth factor, tumor growth factor β 1, platelet derived endothelial cell growth factor, placenta growth factor, and pleitrophin in human primary breast cancer and its relation to angiogenesis. *Cancer Res* **57**: 963–969

Rhoads RE (1993) Regulation of eukaryotic protein synthesis by initiation factors. *J Biol Chem* **266**: 3017–3020

Sacks PM, Smith K, Norman AP, Greenall M, LeJeune S and Harris AL (1993) Cathepsin D levels in primary breast cancers: relationship with epidermal growth factor receptor, oestrogen receptor and axillary node status. *Eur J Cancer* **29**: 426–428

Sato K, Terada K, Sugiyama T, Takahashi S, Saito M, Moriama M, Kikunuma H, Suzuki Y, Kato M and Kao T (1994) Frequent overexpression of vascular endothelial growth-factor gene in human renal-cell carcinoma. *Tohoku J Exp Med* **173**: 355–360

Scott PAE and Harris AL (1994) Current approaches to targeting cancer using antiangiogenesis therapies. *Cancer Treat Rev* **20**: 393–412

Scott PAE, Smith K, Bicknell R and Harris AL (1997) A reliable external control for ribonuclease protection assays. *Nucleic Acids Res* **25**: 1305–1306

Senior PV, Critchley DR, Beck F, Walker RA and Varley JM (1988) The localization of laminin mRNA and protein in the postimplantation embryo and placenta of the mouse: an in situ hybridisation and immunocytochemical study. *Development* **104**: 431–436

Shantz LM, Hu RH and Pegg AE (1996) Regulation of ornithine decarboxylase in a transformed cell line that overexpresses translation intiation factor eIF-4E. *Cancer Res* **56**: 3265–3269

Shimogori T, Suzuki T, Kashiwagi K, Kikunuma Y and Igarashi K (1996) Enhancement of helicase activity and increase of eIF-4E phosphorylation in ornithine decarboxylase-overproducing cells. *Biochem Biophys Res Comm* **222**: 748–752

Shiraishi A, Ishiwata T, Shoji T and Asano G (1995) Expression of PCNA, basic fibroblast growth factor, FGF-receptor and vascular endothelial growth factor in adenomas and carcinomas of human colon. *Acta Histochim Cytochim* **28**: 21–29

Shweiki D, Itin A, Soffer D and Keshet E (1992) Vascular endothelial growth factor induced by hypoxia may mediate hypoxia-initiated angiogenesis. *Nature* **359**: 843–845

Shweiki D, Neman M, Itin A and Keshet E (1995) Induction of vascular endothelial growth-factor expression by hypoxia and by glucose deficiency in multicell spheroids – implications for tumor angiogenesis. *Proc Natl Acad Sci USA* **92**: 768–772

Soker S, Svahn CM and Neufeld G (1993) Vascular endothelial growth factor is inactivated by binding to alpha 2-macroglobulin and the binding is inhibited by heparin. *J Biol Chem* **268**: 7685–7691

Takahaski A, Sasaki H, Kim SH, Tobisu K, Kakizoe T, Tsukamoto T, Kumanoto Y, Sugimura T and Terada M (1994) Markedly increased amounts of messenger RNAs for vascular endothelial growth factor and placenta growth factor in renal cell carcinoma associated with angiogenesis. *Cancer Res* **54**: 4233–4237

Takahaski Y, Kitadai Y, Bucana CD, Bleary KR and Ellis LM (1995) Expression of vascular endothelial growth-factor and its receptor, kdr, correlates with vascularity, metastasis, and proliferation of human colon-cancer. *Cancer Res* **55**: 3964–3968

Tischer E, Mitchell R, Hartman T, Silva M, Gospodarowicz D, Fiddes JC and Abraham JA (1991) The human gene for vascular endothelial growth factor. Multiple protein forms are encoded through alternative exon splicing. *J Biol Chem* **266**: 11947–11954

Toi M, Hoshina T, Takayanagi T and Tominaga T (1994) Association of vascular endothelial growth factor expression with tumor angiogenesis and with early relapse in primary breast cancer. *Jpn J Cancer Res* **85**: 1045–1049

Toi M, Inada K, Hoshina S, Suzuki H, Kondo S and Tominaga T (1995) Vascular endothelial growth-factor and platelet-derived endothelial-cell growth-factor

© Cancer Research Campaign 1998 

are frequently coexpressed in highly vascularized human breast cancer. *Clin Cancer Res* **1**: 961–964

Toi M, Kondo S, Suzuki H, Yamamoto Y, Inada K, Imazawa T, Taniguchi T and Tominaga T (1996) Quantitative-analysis of vascular endothelial growth-factor in primary breast-cancer. *Cancer* **77**: 1101–1106

Unemori EN, Ferrara N, Bauer EA and Amento EP (1992) Vascular endothelial growth factor induces interstitial collagenase expression in human endothelial cells. *J Cell Physiol* **153**: 557–562

Warren RS, Yuan H, Matli MR, Gillett NA and Ferrara N (1995) Regulation by vascular endothelial growth-factor of human colon-cancer tumorigenesis in a mouse model of experimental liver metastasis. *J Clin Invest* **95**: 1789–1797

Weidner N, Folkman J, Pozza F, Bevilacqua P, Allred EA, Moore H, Meli S and Gasparini G (1992) Tumor angiogenesis: a new significant and independent prognostic indicator in early-stage breast carcinoma. *J Natl Cancer Inst* **84**: 1875–1887

Yoshiji H, Gomez DE, Shibuya M and Thorgeirsson UP (1996) Expression of vascular endothelial growth-factor, its receptor, and other angiogenic factors in human breast cancer. *Cancer Res* **56**: 2013–2016

Zhang HT, Craft P, Scott PAE, Ziche M, Weich HA, Harris AL and Bicknell R (1995) Enhancement of tumor growth and vascular density by transfection of vascular endothelial growth factor into MCF-7 human breast carcinoma cells. *J Natl Cancer Inst* **87**: 213–219

*© Cancer Research Campaign 1998*


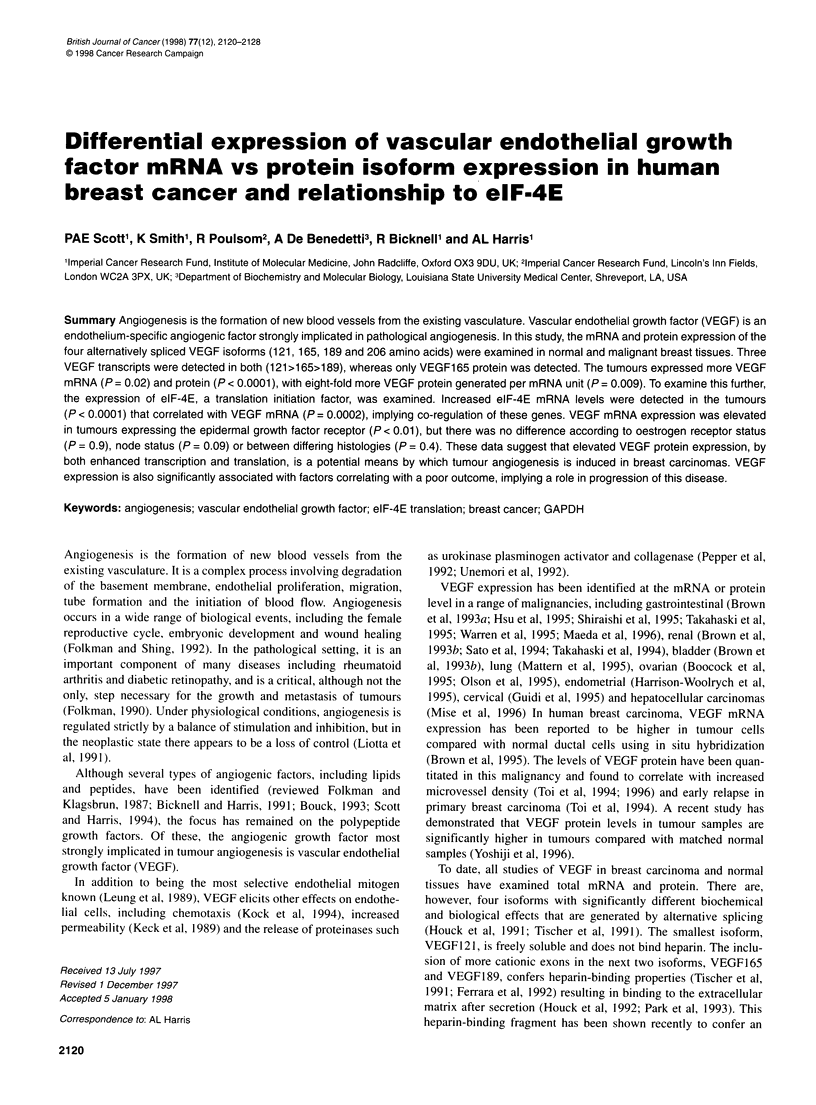

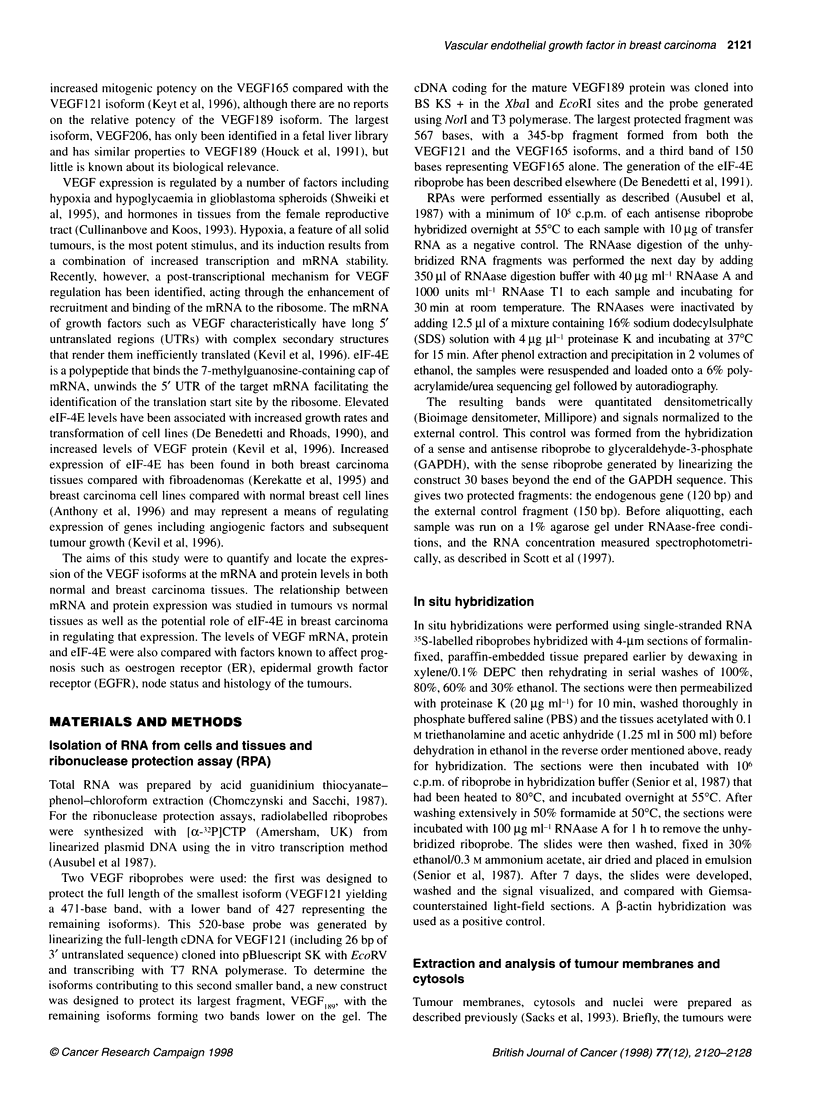

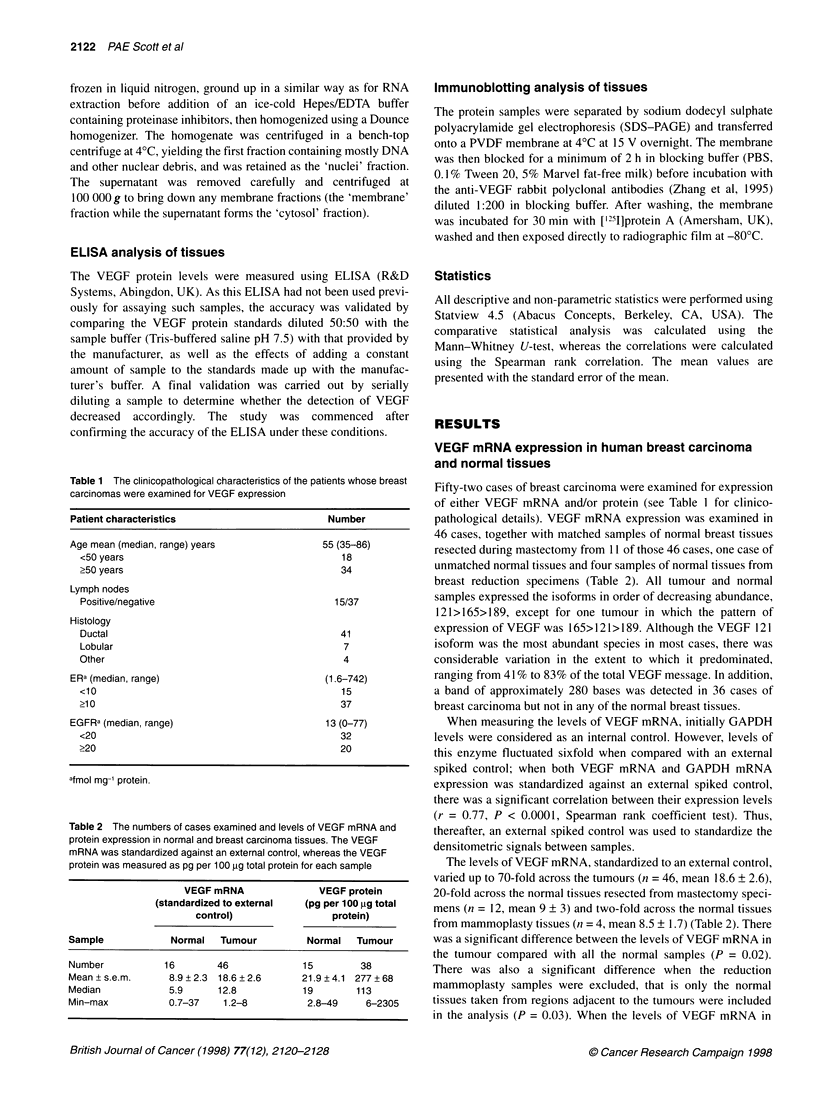

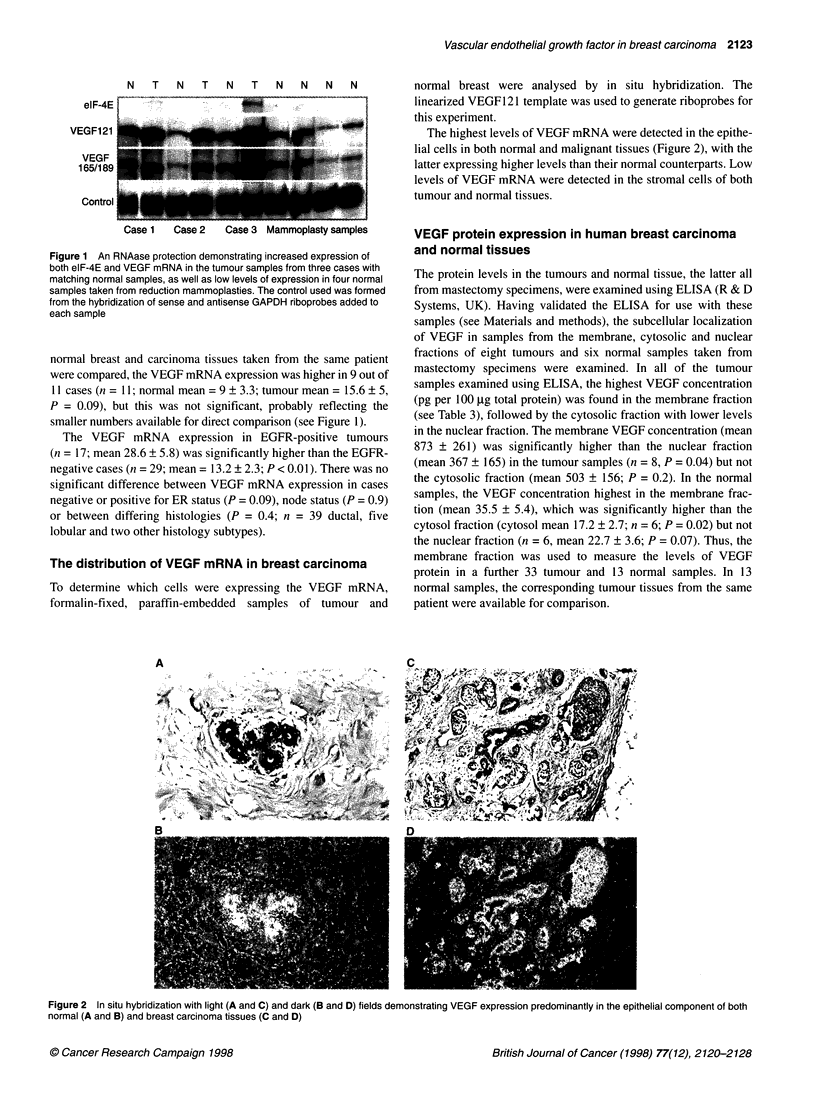

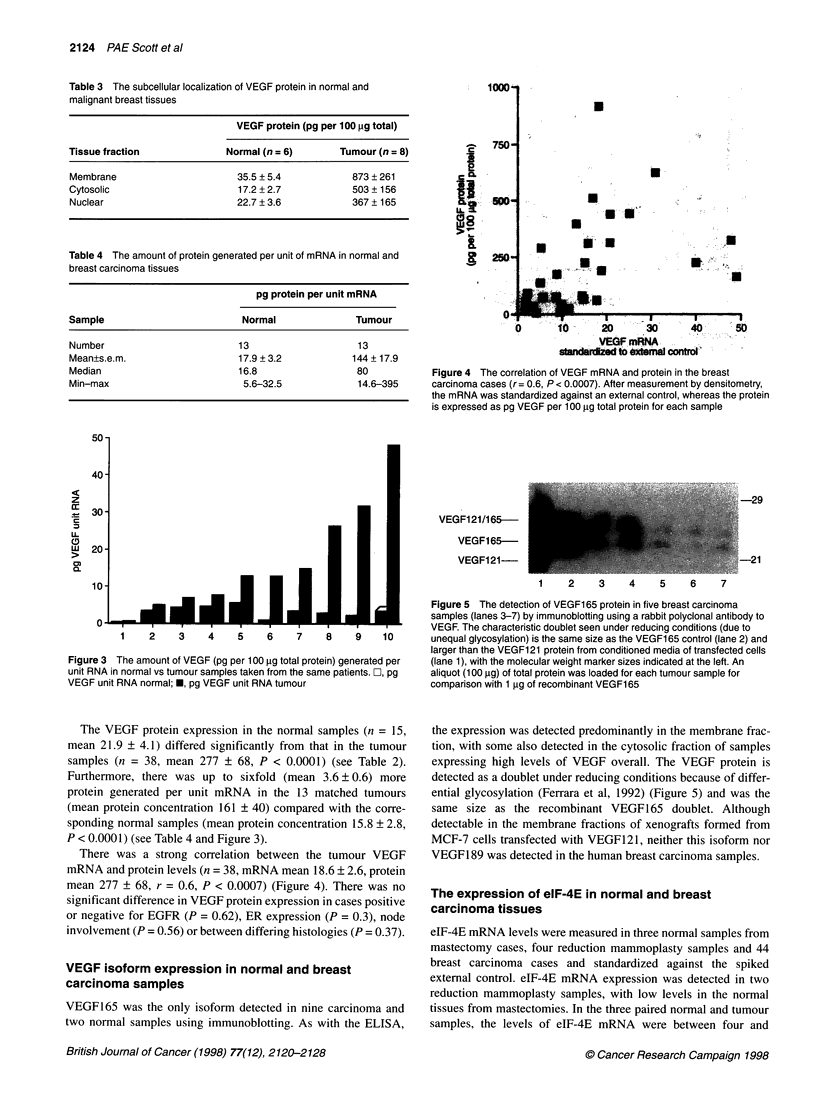

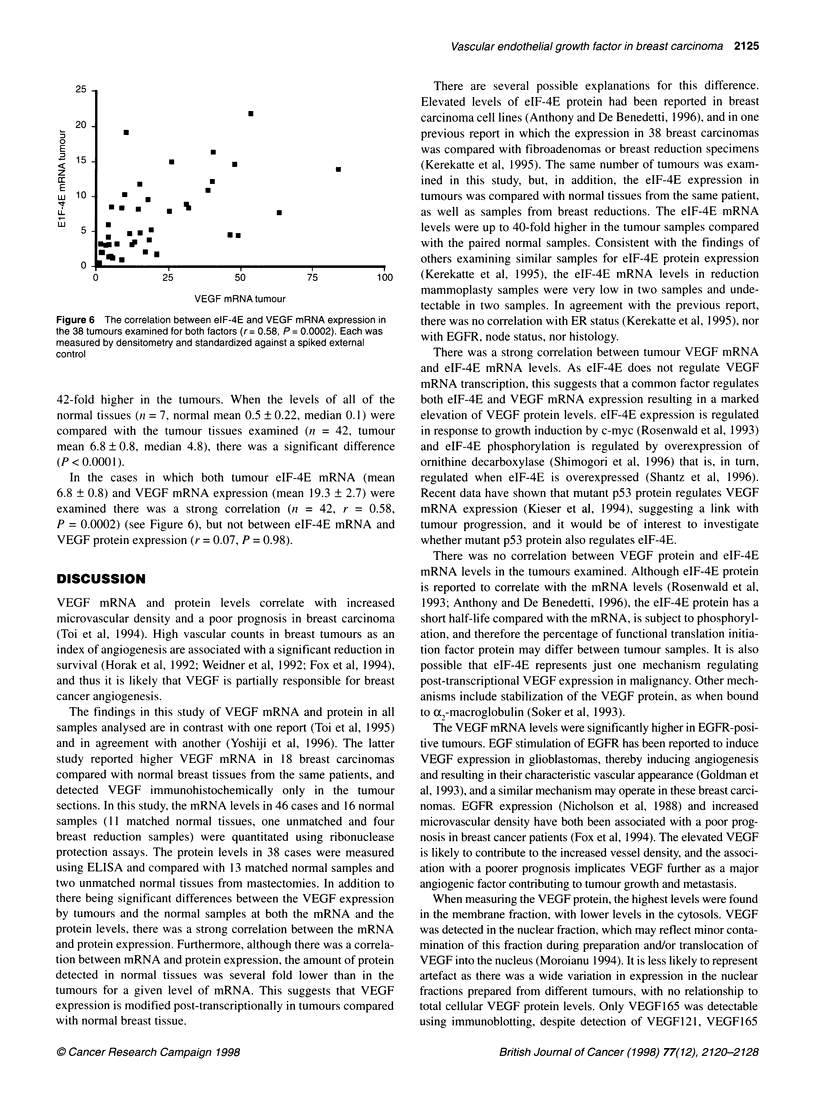

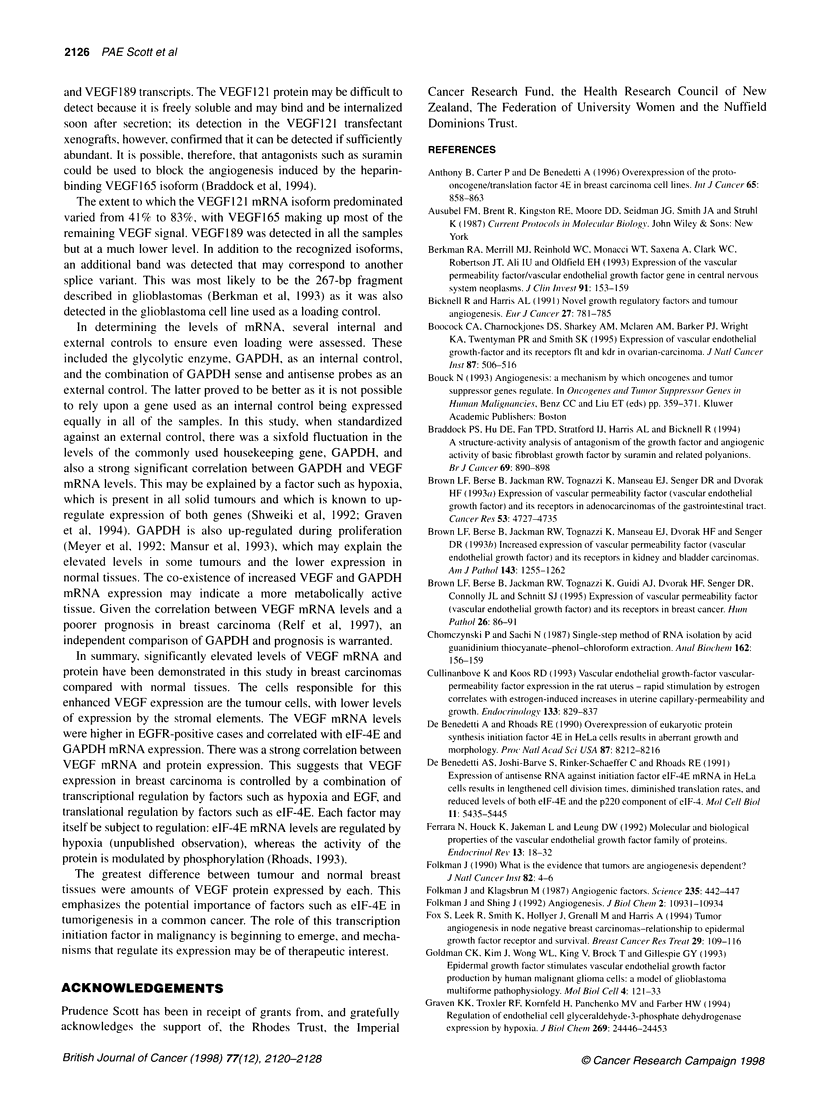

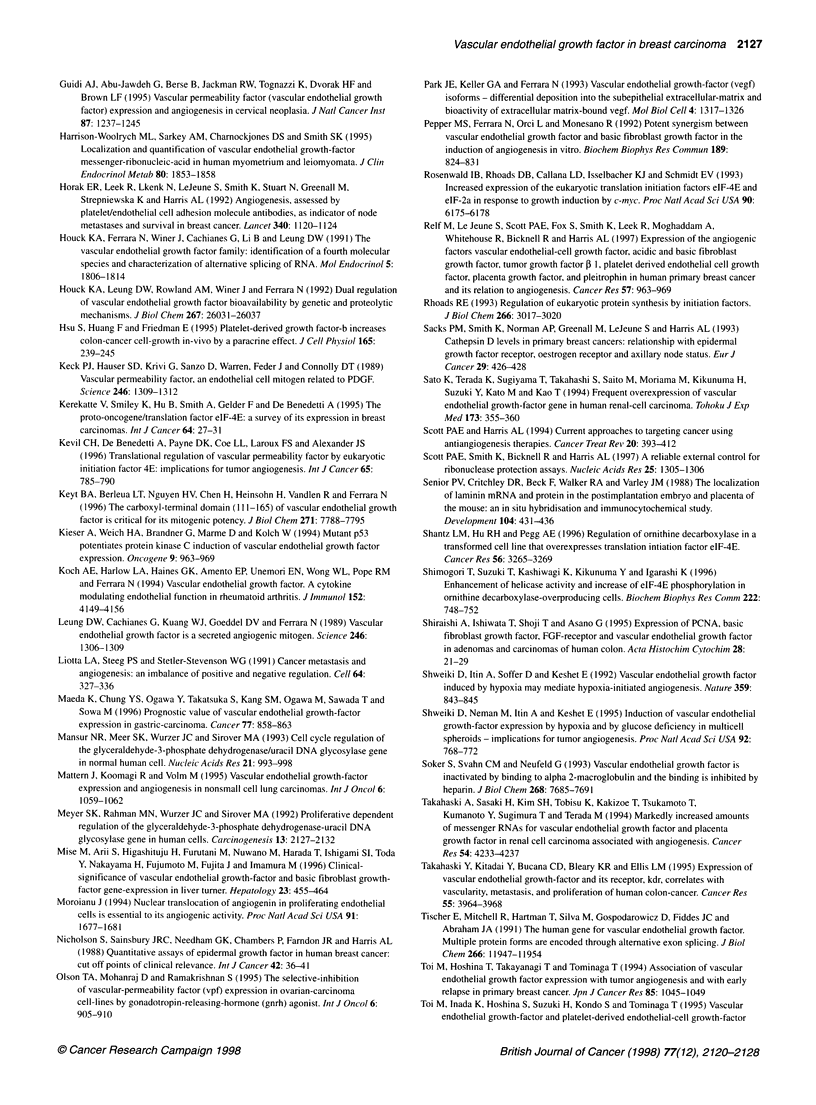

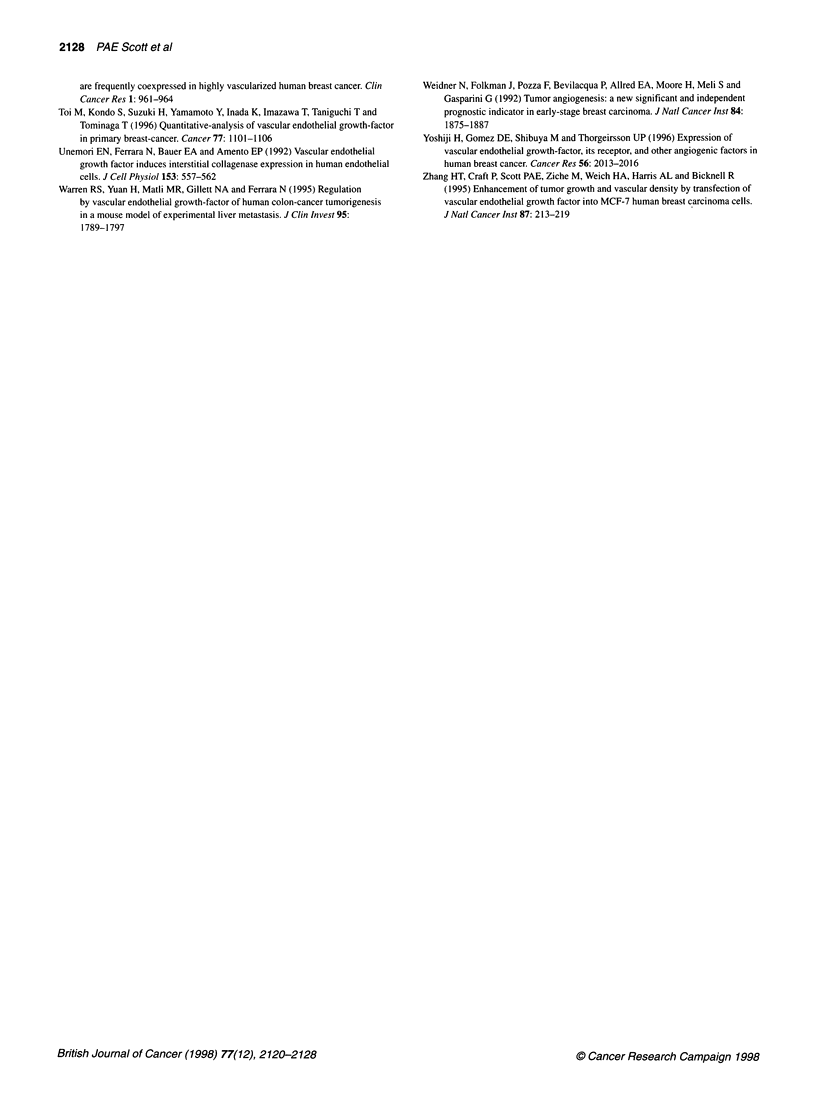


## References

[OCR_00892] Anthony B., Carter P., De Benedetti A. (1996). Overexpression of the proto-oncogene/translation factor 4E in breast-carcinoma cell lines.. Int J Cancer.

[OCR_00904] Berkman R. A., Merrill M. J., Reinhold W. C., Monacci W. T., Saxena A., Clark W. C., Robertson J. T., Ali I. U., Oldfield E. H. (1993). Expression of the vascular permeability factor/vascular endothelial growth factor gene in central nervous system neoplasms.. J Clin Invest.

[OCR_00911] Bicknell R., Harris A. L. (1991). Novel growth regulatory factors and tumour angiogenesis.. Eur J Cancer.

[OCR_00917] Boocock C. A., Charnock-Jones D. S., Sharkey A. M., McLaren J., Barker P. J., Wright K. A., Twentyman P. R., Smith S. K. (1995). Expression of vascular endothelial growth factor and its receptors flt and KDR in ovarian carcinoma.. J Natl Cancer Inst.

[OCR_00946] Brown L. F., Berse B., Jackman R. W., Tognazzi K., Guidi A. J., Dvorak H. F., Senger D. R., Connolly J. L., Schnitt S. J. (1995). Expression of vascular permeability factor (vascular endothelial growth factor) and its receptors in breast cancer.. Hum Pathol.

[OCR_00952] Chomczynski P., Sacchi N. (1987). Single-step method of RNA isolation by acid guanidinium thiocyanate-phenol-chloroform extraction.. Anal Biochem.

[OCR_00957] Cullinan-Bove K., Koos R. D. (1993). Vascular endothelial growth factor/vascular permeability factor expression in the rat uterus: rapid stimulation by estrogen correlates with estrogen-induced increases in uterine capillary permeability and growth.. Endocrinology.

[OCR_00969] De Benedetti A., Joshi-Barve S., Rinker-Schaeffer C., Rhoads R. E. (1991). Expression of antisense RNA against initiation factor eIF-4E mRNA in HeLa cells results in lengthened cell division times, diminished translation rates, and reduced levels of both eIF-4E and the p220 component of eIF-4F.. Mol Cell Biol.

[OCR_00964] De Benedetti A., Rhoads R. E. (1990). Overexpression of eukaryotic protein synthesis initiation factor 4E in HeLa cells results in aberrant growth and morphology.. Proc Natl Acad Sci U S A.

[OCR_00976] Ferrara N., Houck K., Jakeman L., Leung D. W. (1992). Molecular and biological properties of the vascular endothelial growth factor family of proteins.. Endocr Rev.

[OCR_00986] Folkman J., Shing Y. (1992). Angiogenesis.. J Biol Chem.

[OCR_00987] Fox S. B., Leek R. D., Smith K., Hollyer J., Greenall M., Harris A. L. (1994). Tumor angiogenesis in node-negative breast carcinomas--relationship with epidermal growth factor receptor, estrogen receptor, and survival.. Breast Cancer Res Treat.

[OCR_00991] Goldman C. K., Kim J., Wong W. L., King V., Brock T., Gillespie G. Y. (1993). Epidermal growth factor stimulates vascular endothelial growth factor production by human malignant glioma cells: a model of glioblastoma multiforme pathophysiology.. Mol Biol Cell.

[OCR_00997] Graven K. K., Troxler R. F., Kornfeld H., Panchenko M. V., Farber H. W. (1994). Regulation of endothelial cell glyceraldehyde-3-phosphate dehydrogenase expression by hypoxia.. J Biol Chem.

[OCR_01006] Guidi A. J., Abu-Jawdeh G., Berse B., Jackman R. W., Tognazzi K., Dvorak H. F., Brown L. F. (1995). Vascular permeability factor (vascular endothelial growth factor) expression and angiogenesis in cervical neoplasia.. J Natl Cancer Inst.

[OCR_01012] Harrison-Woolrych M. L., Sharkey A. M., Charnock-Jones D. S., Smith S. K. (1995). Localization and quantification of vascular endothelial growth factor messenger ribonucleic acid in human myometrium and leiomyomata.. J Clin Endocrinol Metab.

[OCR_01019] Horak E. R., Leek R., Klenk N., LeJeune S., Smith K., Stuart N., Greenall M., Stepniewska K., Harris A. L. (1992). Angiogenesis, assessed by platelet/endothelial cell adhesion molecule antibodies, as indicator of node metastases and survival in breast cancer.. Lancet.

[OCR_01026] Houck K. A., Ferrara N., Winer J., Cachianes G., Li B., Leung D. W. (1991). The vascular endothelial growth factor family: identification of a fourth molecular species and characterization of alternative splicing of RNA.. Mol Endocrinol.

[OCR_01033] Houck K. A., Leung D. W., Rowland A. M., Winer J., Ferrara N. (1992). Dual regulation of vascular endothelial growth factor bioavailability by genetic and proteolytic mechanisms.. J Biol Chem.

[OCR_01038] Hsu S., Huang F., Friedman E. (1995). Platelet-derived growth factor-B increases colon cancer cell growth in vivo by a paracrine effect.. J Cell Physiol.

[OCR_01043] Keck P. J., Hauser S. D., Krivi G., Sanzo K., Warren T., Feder J., Connolly D. T. (1989). Vascular permeability factor, an endothelial cell mitogen related to PDGF.. Science.

[OCR_01048] Kerekatte V., Smiley K., Hu B., Smith A., Gelder F., De Benedetti A. (1995). The proto-oncogene/translation factor eIF4E: a survey of its expression in breast carcinomas.. Int J Cancer.

[OCR_01053] Kevil C. G., De Benedetti A., Payne D. K., Coe L. L., Laroux F. S., Alexander J. S. (1996). Translational regulation of vascular permeability factor by eukaryotic initiation factor 4E: implications for tumor angiogenesis.. Int J Cancer.

[OCR_01059] Keyt B. A., Berleau L. T., Nguyen H. V., Chen H., Heinsohn H., Vandlen R., Ferrara N. (1996). The carboxyl-terminal domain (111-165) of vascular endothelial growth factor is critical for its mitogenic potency.. J Biol Chem.

[OCR_01063] Kieser A., Weich H. A., Brandner G., Marmé D., Kolch W. (1994). Mutant p53 potentiates protein kinase C induction of vascular endothelial growth factor expression.. Oncogene.

[OCR_01068] Koch A. E., Harlow L. A., Haines G. K., Amento E. P., Unemori E. N., Wong W. L., Pope R. M., Ferrara N. (1994). Vascular endothelial growth factor. A cytokine modulating endothelial function in rheumatoid arthritis.. J Immunol.

[OCR_01075] Leung D. W., Cachianes G., Kuang W. J., Goeddel D. V., Ferrara N. (1989). Vascular endothelial growth factor is a secreted angiogenic mitogen.. Science.

[OCR_01080] Liotta L. A., Steeg P. S., Stetler-Stevenson W. G. (1991). Cancer metastasis and angiogenesis: an imbalance of positive and negative regulation.. Cell.

[OCR_01085] Maeda K., Chung Y. S., Ogawa Y., Takatsuka S., Kang S. M., Ogawa M., Sawada T., Sowa M. (1996). Prognostic value of vascular endothelial growth factor expression in gastric carcinoma.. Cancer.

[OCR_01090] Mansur N. R., Meyer-Siegler K., Wurzer J. C., Sirover M. A. (1993). Cell cycle regulation of the glyceraldehyde-3-phosphate dehydrogenase/uracil DNA glycosylase gene in normal human cells.. Nucleic Acids Res.

[OCR_01100] Meyer-Siegler K., Rahman-Mansur N., Wurzer J. C., Sirover M. A. (1992). Proliferative dependent regulation of the glyceraldehyde-3-phosphate dehydrogenase/uracil DNA glycosylase gene in human cells.. Carcinogenesis.

[OCR_01105] Mise M., Arii S., Higashituji H., Furutani M., Niwano M., Harada T., Ishigami S., Toda Y., Nakayama H., Fukumoto M. (1996). Clinical significance of vascular endothelial growth factor and basic fibroblast growth factor gene expression in liver tumor.. Hepatology.

[OCR_01112] Moroianu J., Riordan J. F. (1994). Nuclear translocation of angiogenin in proliferating endothelial cells is essential to its angiogenic activity.. Proc Natl Acad Sci U S A.

[OCR_01117] Nicholson S., Sainsbury J. R., Needham G. K., Chambers P., Farndon J. R., Harris A. L. (1988). Quantitative assays of epidermal growth factor receptor in human breast cancer: cut-off points of clinical relevance.. Int J Cancer.

[OCR_01129] Park J. E., Keller G. A., Ferrara N. (1993). The vascular endothelial growth factor (VEGF) isoforms: differential deposition into the subepithelial extracellular matrix and bioactivity of extracellular matrix-bound VEGF.. Mol Biol Cell.

[OCR_01133] Pepper M. S., Ferrara N., Orci L., Montesano R. (1992). Potent synergism between vascular endothelial growth factor and basic fibroblast growth factor in the induction of angiogenesis in vitro.. Biochem Biophys Res Commun.

[OCR_01145] Relf M., LeJeune S., Scott P. A., Fox S., Smith K., Leek R., Moghaddam A., Whitehouse R., Bicknell R., Harris A. L. (1997). Expression of the angiogenic factors vascular endothelial cell growth factor, acidic and basic fibroblast growth factor, tumor growth factor beta-1, platelet-derived endothelial cell growth factor, placenta growth factor, and pleiotrophin in human primary breast cancer and its relation to angiogenesis.. Cancer Res.

[OCR_01154] Rhoads R. E. (1993). Regulation of eukaryotic protein synthesis by initiation factors.. J Biol Chem.

[OCR_01139] Rosenwald I. B., Rhoads D. B., Callanan L. D., Isselbacher K. J., Schmidt E. V. (1993). Increased expression of eukaryotic translation initiation factors eIF-4E and eIF-2 alpha in response to growth induction by c-myc.. Proc Natl Acad Sci U S A.

[OCR_01158] Sacks N. P., Smith K., Norman A. P., Greenall M., LeJeune S., Harris A. L. (1993). Cathepsin D levels in primary breast cancers: relationship with epidermal growth factor receptor, oestrogen receptor and axillary nodal status.. Eur J Cancer.

[OCR_01164] Sato K., Terada K., Sugiyama T., Takahashi S., Saito M., Moriyama M., Kakinuma H., Suzuki Y., Kato M., Kato T. (1994). Frequent overexpression of vascular endothelial growth factor gene in human renal cell carcinoma.. Tohoku J Exp Med.

[OCR_01171] Scott P. A., Harris A. L. (1994). Current approaches to targeting cancer using antiangiogenesis therapies.. Cancer Treat Rev.

[OCR_01175] Scott P. A., Smith K., Bicknel R., Harris A. L. (1997). A reliable external control for ribonuclease protection assays.. Nucleic Acids Res.

[OCR_01179] Senior P. V., Critchley D. R., Beck F., Walker R. A., Varley J. M. (1988). The localization of laminin mRNA and protein in the postimplantation embryo and placenta of the mouse: an in situ hybridization and immunocytochemical study.. Development.

[OCR_01185] Shantz L. M., Hu R. H., Pegg A. E. (1996). Regulation of ornithine decarboxylase in a transformed cell line that overexpresses translation initiation factor eIF-4E.. Cancer Res.

[OCR_01190] Shimogori T., Suzuki T., Kashiwagi K., Kakinuma Y., Igarashi K. (1996). Enhancement of helicase activity and increase of eIF-4E phosphorylation in ornithine decarboxylase-overproducing cells.. Biochem Biophys Res Commun.

[OCR_01203] Shweiki D., Itin A., Soffer D., Keshet E. (1992). Vascular endothelial growth factor induced by hypoxia may mediate hypoxia-initiated angiogenesis.. Nature.

[OCR_01208] Shweiki D., Neeman M., Itin A., Keshet E. (1995). Induction of vascular endothelial growth factor expression by hypoxia and by glucose deficiency in multicell spheroids: implications for tumor angiogenesis.. Proc Natl Acad Sci U S A.

[OCR_01215] Soker S., Svahn C. M., Neufeld G. (1993). Vascular endothelial growth factor is inactivated by binding to alpha 2-macroglobulin and the binding is inhibited by heparin.. J Biol Chem.

[OCR_01220] Takahashi A., Sasaki H., Kim S. J., Tobisu K., Kakizoe T., Tsukamoto T., Kumamoto Y., Sugimura T., Terada M. (1994). Markedly increased amounts of messenger RNAs for vascular endothelial growth factor and placenta growth factor in renal cell carcinoma associated with angiogenesis.. Cancer Res.

[OCR_01228] Takahashi Y., Kitadai Y., Bucana C. D., Cleary K. R., Ellis L. M. (1995). Expression of vascular endothelial growth factor and its receptor, KDR, correlates with vascularity, metastasis, and proliferation of human colon cancer.. Cancer Res.

[OCR_01235] Tischer E., Mitchell R., Hartman T., Silva M., Gospodarowicz D., Fiddes J. C., Abraham J. A. (1991). The human gene for vascular endothelial growth factor. Multiple protein forms are encoded through alternative exon splicing.. J Biol Chem.

[OCR_01241] Toi M., Hoshina S., Takayanagi T., Tominaga T. (1994). Association of vascular endothelial growth factor expression with tumor angiogenesis and with early relapse in primary breast cancer.. Jpn J Cancer Res.

[OCR_01246] Toi M., Inada K., Hoshina S., Suzuki H., Kondo S., Tominaga T. (1995). Vascular endothelial growth factor and platelet-derived endothelial cell growth factor are frequently coexpressed in highly vascularized human breast cancer.. Clin Cancer Res.

[OCR_01257] Toi M., Kondo S., Suzuki H., Yamamoto Y., Inada K., Imazawa T., Taniguchi T., Tominaga T. (1996). Quantitative analysis of vascular endothelial growth factor in primary breast cancer.. Cancer.

[OCR_01262] Unemori E. N., Ferrara N., Bauer E. A., Amento E. P. (1992). Vascular endothelial growth factor induces interstitial collagenase expression in human endothelial cells.. J Cell Physiol.

[OCR_01267] Warren R. S., Yuan H., Matli M. R., Gillett N. A., Ferrara N. (1995). Regulation by vascular endothelial growth factor of human colon cancer tumorigenesis in a mouse model of experimental liver metastasis.. J Clin Invest.

[OCR_01273] Weidner N., Folkman J., Pozza F., Bevilacqua P., Allred E. N., Moore D. H., Meli S., Gasparini G. (1992). Tumor angiogenesis: a new significant and independent prognostic indicator in early-stage breast carcinoma.. J Natl Cancer Inst.

[OCR_01279] Yoshiji H., Gomez D. E., Shibuya M., Thorgeirsson U. P. (1996). Expression of vascular endothelial growth factor, its receptor, and other angiogenic factors in human breast cancer.. Cancer Res.

[OCR_01284] Zhang H. T., Craft P., Scott P. A., Ziche M., Weich H. A., Harris A. L., Bicknell R. (1995). Enhancement of tumor growth and vascular density by transfection of vascular endothelial cell growth factor into MCF-7 human breast carcinoma cells.. J Natl Cancer Inst.

